# Enabling Community Through Social Media

**DOI:** 10.2196/jmir.2796

**Published:** 2013-10-31

**Authors:** Anatoliy Gruzd, Caroline Haythornthwaite

**Affiliations:** ^1^School of Information ManagementFaculty of ManagementDalhousie UniversityHalifax, NSCanada; ^2^School of Library, Archival and Information StudiesThe iSchool at UBCUniversity of British ColumbiaVancouver, BCCanada

**Keywords:** online community, online social networks, information and communication technology, social media, Twitter

## Abstract

**Background:**

Social network analysis provides a perspective and method for inquiring into the structures that comprise online groups and communities. Traces from interaction via social media provide the opportunity for understanding how a community is formed and maintained online.

**Objective:**

The paper aims to demonstrate how social network analysis provides a vocabulary and set of techniques for examining interaction patterns via social media. Using the case of the #hcsmca online discussion forum, this paper highlights what has been and can be gained by approaching online community from a social network perspective, as well as providing an inside look at the structure of the #hcsmca community.

**Methods:**

Social network analysis was used to examine structures in a 1-month sample of Twitter messages with the hashtag #hcsmca (3871 tweets, 486 unique posters), which is the tag associated with the social media–supported group Health Care Social Media Canada. Network connections were considered present if the individual was mentioned, replied to, or had a post retweeted.

**Results:**

Network analyses revealed patterns of interaction that characterized the community as comprising one component, with a set of core participants prominent in the network due to their connections with others. Analysis showed the social media health content providers were the most influential group based on in-degree centrality. However, there was no preferential attachment among people in the same professional group, indicating that the formation of connections among community members was not constrained by professional status.

**Conclusions:**

Network analysis and visualizations provide techniques and a vocabulary for understanding online interaction, as well as insights that can help in understanding what, and who, comprises and sustains a network, and whether community emerges from a network of online interactions.

## Introduction

### Background

The use of social media has spread dramatically in the past few years, demonstrated in increasing numbers of users, types of media, mobile applications, and connectivity. This has stimulated growth in applying social media to matters of health and health communities: from work-based communities of practice [1] to forums for patient social and information support (eg, [2-11]). These efforts can be enhanced by taking advantage of the research and experience already existing relating to online communication and community. This research provides a wealth of background theories, studies, and findings that inform the ways that a community is likely to form via newer social media and that can be applied to the development of health communities.

Of the many approaches to community and online communication that have emerged, we highlight a social network perspective. This perspective looks at group or community interactions to determine what kinds of actors and ties make up the network; what exchange of information, social support, socializing, play, or other resources form the basis of the community; and what roles and cliques emerge that provide structure to the community. Social network analysis provides a vocabulary and set of techniques for examining interaction patterns between people and has proven useful for studying health (eg, [12,13]), how relationships are maintained without physical co-presence [14-17], and the development of new, health care-related online networks [3].

In this paper, we first discuss social networks and then illustrate the kind of information that can be revealed about community from a social network perspective through a case study of social media use by the group Health Care Social Media Canada (HCSMCA). This case includes network analyses of the group’s structures as shown through a sample of Twitter messages using the hashtag #hcsmca. Results from the network analysis reveal a cohesive group consisting of one major component, including interaction across professional roles. The group founder and participants that are identified as social media health care providers are prominent in posts and in attention from others, and the network is sustained by participation from and recognition of a core set of actors.

The first section below reviews the background on online social networks and describes the HCSMCA group. The following sections describe the analysis of the #hcsmca Twitter networks and then discuss these in relation to previous research on online communities.

### Social Network Analysis

Of the many ways to look at the range and effects of social media on interpersonal and collective relations, one that has proved useful for online communities has been a social network perspective. This is not the same as “social networking”. It is instead an approach that considers the unit of analysis to be the connections between people and looks at how these connections—social network “relations”—form patterns of interaction that reveal how information and other resources flow in a network, as well as the structures that define the network [18]. Pescosolido [19] has suggested that a network-centered view of health, based on social network principles, can act as a bridge between medical sciences and individual health experience. Her ideas respond to an increasing recognition of the impact of connectivity and experience: “The individual is seen as embedded in an ongoing relational dynamic with sequences of events seen as patterned, contingent and emergent” (p. 196). Her network episode model makes a connection between social context, social support, and illness careers and offers a way to address the complex whole that pertains to health and well-being.

Media use is just one aspect of this complex structure, but it has the potential to set context, add to a social support system, and touch individuals and their closely tied friends and family. From an analytical perspective, one of the advantages of taking a social network perspective is that the focus is on what people do with each other rather than the medium or face-to-face context through which they do it. This allows exploration of the types of interactions that create and define different kinds of relationships and communities [20]. Thus, friendship may be recognized by pairwise exchange of personal information and emotion, discussion of multiple topics, co-participation in events, frequent interaction, and the use of multiple media. Social support emerges as a complex of small and larger exchanges between people, trust in networks to provide services in time of need, and a generalized reciprocity in communities where resources are distributed more generally than in a strictly give-and-take fashion. Analyzing health support networks requires understanding not just what media are used for communication, but also the types of exchanges that constitute support and the roles that start a network, as well as the ones that emerge from networks.

Many years of research on social networks have provided evidence of social network principles as well as statistical and analytical techniques for understanding network behaviors (eg, [21-24]), including health [19,12,25]. The basic principles of social network analysis are derived from graph theory and consider *actors* (eg, people, organizations) as nodes in a network, connected by *relations* (what they do with each other, eg, provide new information, emotional support, resources, and/or services) that form interpersonal *ties*. The nature and variety of relations define the kind of relationship between actors, such as an acquaintanceship, friendship, learning, or work relationship. Research has shown that the closer the relationship, the more different types of exchanges are maintained and the more important these exchanges are for the individuals; close personal relationships also demonstrate a higher level of intimacy and self-disclosure. Such ties are *strong ties*, and pairs who are strongly tied are more motivated to share their resources with each other. These pairs also turn out to be more like each other (more homophilous), with the result that they tend to know and associate with similar others. *Weak ties*, by contrast, are less motivated to share their resources but are more likely to have access to resources different from each other because they do not share similar habits, circles of friends, etc [26]. Pairwise relationships build into the social networks that are recognized as cliques, groups, and communities.

Where bonds are strong, resources are shared generally around the network (generalized reciprocity). This creates the social capital of the network, that is, the accumulated resources held within the network rather than those held by any individual [27]. Where bonds link networks, they connect the network to resources in other networks. Putnam [28] describes these two forms as *bonding* and *bridging social capital*. Both are important means of information and resource access and uptake. Burt [29] identified the important position of the actor who acts as a broker between networks, filling a *structural hole*. Such an actor can choose to control information and resources between these two separate networks, or they can facilitate its transmission. Recently the latter position has come to prominence embodied in the role of the “social entrepreneur”, that is, an individual positioned to facilitate the transfer of knowledge or practices to disadvantaged—non-networked—communities (eg, [30]).

The configuration of connections is all-important in social networks. These structures show how actors are connected over the whole network, and thus what paths and obstacles there are for contact, information, and resource flow. Among popular aspects considered for networks are the *positions* of individuals, for example, how prominent or influential they can be based on the ties to and from other actors (creating recognized positions such as network stars, isolates, brokers). For networks as a whole, cliques may be evident as highly interconnected subsets of network actors. Networks may exhibit a high or low density of internal connections, with the former suggesting rapid diffusion of resources and the latter suggesting slow, poor, or long-chain routes for diffusion. Also of interest, particularly when comparing across networks, are similarities in structures and *roles*, for example, as a teacher fills the same role with students no matter what class is examined, or a doctor with a patient no matter what the medical setting.

Our online interactions make these patterns more readily observable, and many examples exist now of how such patterns can be made visible, for example, in social network interaction patterns [31], patterns of text changes in wikis [32,33], and information seeking patterns (eg, Google Flu trends), each of which contributes to understanding emergent community network properties [34]. Social media traces are thus an entry point to describing and later understanding and facilitating community interaction.

In this paper, we examine the social media traces from the #hcsmca Twitter posts. We examine what social network patterns are revealed and the implications these have for #hcsmca as a community. The following section provides background on the #hcsmca group and its operation.

### #hcsmca—A Twitter Community

As stated on the HCSMCA website, “#hcsmca is a vibrant community of people interested in exploring social innovation in health care. We share and learn, and together we are making health care more open and connected.” It is an example of how those with a common interest can meet and form community online through social media, in this case, in the interests of social innovation in Canadian health care.

The community was founded in September 2010, by Colleen Young, an online community manager and Toronto-based patient advocate and health writer [35]. In her blog [36], she describes the community as follows:

Anyone and everyone delivering and receiving health care who is interested in open conversation to help improve quality, access, value and effectiveness of health care. This includes: patients, caregivers, patient advocates, health care professionals, not-for-profit health organizations, educators, health content providers, health institutions, health administrators, health systems and networks, government and health policy makers.

The community is maintained through four social media: Twitter, a LinkedIn group with 181 members, a Facebook page with 143 “likes” (as of January 8, 2013), and the blog maintained by the founder, Colleen Young. While maintained across these various media, the community relies on Twitter, the popular microblogging site, as their primary communication platform, operating with the hashtag #hcsmca. The community meets weekly on Twitter to discuss various topics relating to health care and social media.

To participate in this group discussion, a participant just needs to post a message on Twitter using this hashtag. At the time of this research, weekly chats on Twitter are scheduled for every Wednesday at 1 pm EST with the last Wednesday of the month being an evening chat at 9 pm EST. Weekly topics and guest moderators are announced in advance and listed in a public Google spreadsheet. For those who miss this real-time meeting, a transcript with messages is available, posted to the community blog by the group moderator. #hcsmca is a great case for study since HCSMCA has been active with this hashtag for over 2 years and generates very active weekly discussions that attract a wide variety of professionals and organizations.

One of the main goals of our research is to gain a better understanding of how social media–based information and communication technologies, such as Twitter, enable a distributed group of people to form and maintain an online community. In particular, we are interested in the following research questions regarding #hcsmca:

1. What accounts for the relative longevity of this particular online community? Is it because of the founder’s leadership and continuing involvement, or are there core members who are actively and persistently involved in this community?
2. What is the composition of this community in general? And, more specifically, does their professional role determine a person’s centrality within this community? This will allow us to understand generally how professional roles affect online conversational dynamics, and more specifically whether this online community is a welcoming place for a wide range of professionals or is, instead, dominated by professionals from a particular group.

## Methods

### Study Sample

The primary dataset for this research came from Twitter and included all public Twitter messages that included the #hcsmca hashtag, posted between November 12, 2012, and December 13, 2012. The dataset contains a total of 3871 tweets, posted by 486 unique Twitter users. The dataset was collected and analyzed using Netlytic [37] system for automated collection and analysis of social media data. (Netlytic is developed and maintained by author Gruzd).

**Figure 1:**
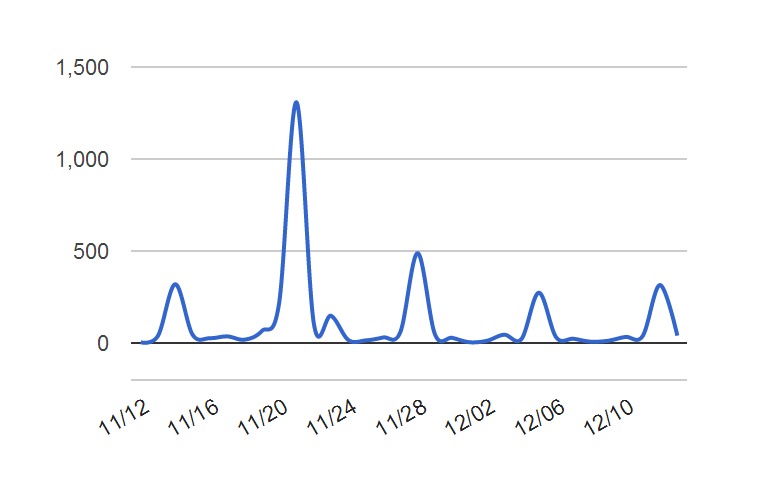
Number of #hcsmca tweets over the studied period.

As noted above, #hcsmca hosts a weekly discussion. Topics covered and the assigned topic moderators for the period studied are shown in Table 1. Figure 1 shows the distribution of the messages over the studied time period; peaks on the chart reflect the weekly live chats.

**Table 1:** #hcsmca weekly topics (Nov 12-Dec 13, 2012).

Date	Weekly discussion topics	Assigned moderator
Nov 14	Challenge of engaging SM [social media] to inform a research agenda	@QuintePediatric
	Use of innovation, SM, and gamification to encourage uptake of self-care	
Nov 21	Health care blogs should we or shouldn’t we, what have we learned, what are the benefits?	@JackieHickeyRN
	Are health care blogs a useful tool for education and knowledge transfer?	
Nov 28	How has social media made you healthier? Unhealthier? Has social media made our health choices more numerous and this overwhelming?	@NaheedD
	What messaging would motivate you to make a positive health change? Who would you listen to?	
Dec 5	What is needed to make cross-organizational collaboration via social networks more effective?	@WillFalk & @MarkCasselman
	In what settings / sectors are you seeing health care providers and patients interacting via social media?	
Dec 12	How can SM support patient care in an ambulatory care setting?	@CraigTyyz
	How can SM help patients/families navigate a new/unfamiliar hospital/clinic/facility?	

### Twitter Networks

Twitter connections are maintained through the technical means of *usernames*, *following*, and *hashtags*. Twitter usernames identify nodes in the network (eg, author Gruzd is identified as “dalprof”). A direct communication connection can be made person to person by indicating the one recipient by prefacing the message—or tweet—with “@” and the username (eg, @dalprof), or tweets can be sent to the world at large. An indirect communication connection can be made by simply mentioning someone’s Twitter username (prefacing it with @) anywhere in a tweet or publicly reposting (retweeting) somebody’s else tweet. (While we say “person to person”, usernames are also commonly associated with groups or organizations; also, no one-to-one correspondence of person to username is assumed as individuals may have several Twitter usernames.)

*Follow* and topic hashtags show relational connections between nodes. Searching for someone on Twitter brings up the option to follow that person’s postings, with their tweets immediately visible on the user’s home Twitter page. *Following* is a node-to-node connection, marking social networks created through the act of designating a *follow* relation in Twitter. A second technical feature for relational connections is the use of hashtags. A hashtag is a microblogging convention that allows users to see others’ messages regardless of whether they have chosen to follow that person. When many people tweet with a common hashtag, this creates connections among posts based on a common hashtag relation. For example, the hashtag #med2 was used at the 2013 Medicine 2.0 conference in London, England. Participants both in London and elsewhere could monitor messages with this hashtag to engage with the Twitter conversation regarding the conference. Both following and hashtags provide the infrastructure for social networks, that is, the underpinning structure from which and on which communities grow and prosper.

### Analyzing Posts for Name Networks

Netlytic was used to discover the communication network among community members. In particular, to discover social connections among community members, the analysis relied on a type of network called “Name Network” [38]. The Name Network technique examines the content of the messages and connects one person to another if they mention, reply, or repost another person’s tweet [39,40]. The resulting network generated by Netlytic included 486 nodes and 736 ties. The collected social network dataset was then exported to the network visualization application ORA [41] and to Ucinet [42] for statistical tests.

**Figure 2:**
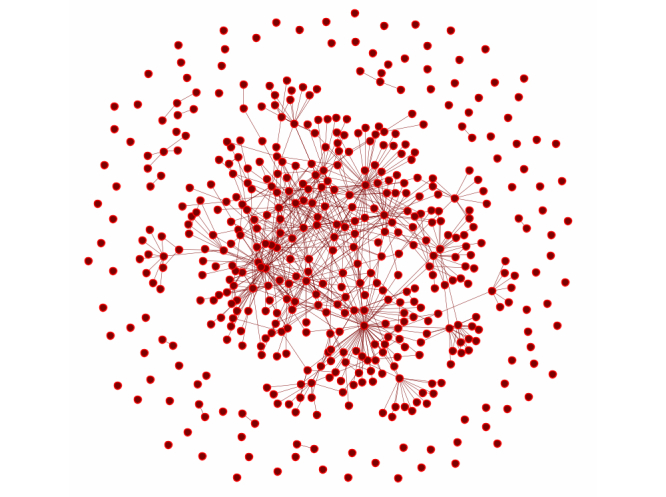
Twitter communication network among #hcsmca participants.

Figure 2 presents the visualization of the #hcsmca Name Network for the 4-week period. The overall view shows a fairly densely connected, single component of posters who are reading and responding to each others’ posts, suggesting an engaged community, paying attention to the topic and actively conversing around the common topic. Isolated nodes (those with no line connecting to others) posted but received no mention, reply, or repost. While there are number of such nodes, their numbers do not overwhelm the number in the central component. Such “legitimate peripheral participation” [43] is a common part of any community and supports learning the way to engage in a community as well as engaging in a partial way that fits individuals’ time and needs. Noticeably absent from this figure are subcliques that carry on side conversations with each other. This shows that the #hcsmca community is not fractionated, but rather that participants are all engaged with the single conversational network.

The following sections show results from the analysis of the Twitter posts, with attention to aspects of community. Results address first, the discovery of key actors in the network and their potential influence on others and second, whether and how professional roles affect participation.

## Results

### Discovering Community Leaders

One way to learn how an online community operates is to find out about key members who have the potential to influence tone, topic, or policy for the whole community. A community organizer may be one such actor, but for a community to operate robustly, actions associated with keeping the community or conversation going need to be distributed to more than one person. Thus, in examining the #hcsmca community, it is of particular interest to see whether more than one individual is leading the discussion.

A brief examination of the community blog shows that, as expected, the founder of the #hcsmca group is heavily involved in planning and running the community. But who else is involved? Are there other members of this group who also take on a leadership role? This is important because the presence of a strong community core with a number of active members suggests a healthy online community that can persist without the presence of particular individuals (eg, as in the failure case described in [44]). For example, if some of the active members cannot participate in a particular weekly discussion, there would be others to carry the conversation.

Three social network measures were used to locate influential individuals in this community: (1) the total number of messages contributed during the studied period, (2) the number of times a person is mentioned or replied to, that is, their @username is used in a post by someone else (*in-degree centrality)*, and (3) the number of times a person mentions or replies to others, that is, an individual uses another person’s @username in a post (*out-degree centrality).*

All three measures are important in identifying prominent individuals in the community. An individual posting a high number of messages gains attention for the content they send to others and can add to the social capital of the community by bringing new information to the group as a whole. However, such information needs to be taken up and used by the community. Thus, a high number of posts, by itself, does not mean that the messages contributed are deemed important or interesting by other members of the community—hence the need to look at network structures of message uptake.

Any use of @username signifies a direct connection between the sender and another individual. Being mentioned by others is a case of *in-degree centrality* and signifies the prestige given to that individual by others in the network. A person mentioning or replying to others indicates *out-degree centrality* and signifies the influence that person has as they make their views known to others. To identify people on Twitter with high *in-degree* centrality values, we look for people whose tweets are chosen by others to be retweeted (forwarded) and/or replied to by many others. To identify people with high *out-degree* centrality on Twitter, we measured how often a person mentioned others or replied to others in their tweets. People who have high out-degree centrality tend to have a good awareness of the network and often monitor and retweet messages by others.

### Total Number of Posts

**Figure 3:**
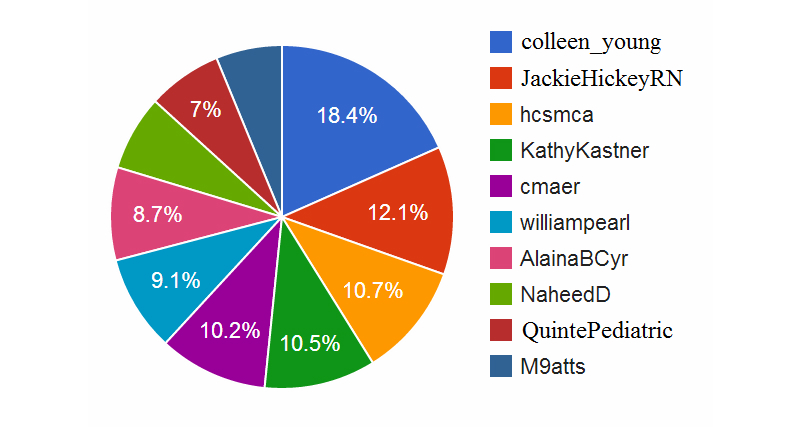
Top 10 most active posters.

Figure 3 shows the top 10 active members of this community based on the total number of messages posted to this community. Not surprisingly, the group organizer, @colleen_young, posted the most number of messages (18.4% of all messages posted by the top 10 posters). In starting an online community, leaders play a key role by their altruistic or proactive participation, providing more posts to the community than they receive and thus helping create a critical mass of interactions that act as a draw for others.

As well as the founder, there are a few other active participants who contribute heavily to the community, posting about the same number of messages each (approximately 10% of the messages posted by the top 10 posters all together). Among this group are people who moderated weekly chats, such as @JackieHickeyRN, @NaheedD, and @QuintePediatric. Such actors also contribute to the critical mass of the conversation, but the more important result is that there are several people the community can rely on to keep the conversation going, increasing the robustness of the ongoing activity.

However, not all of the moderators are active posters. For example, 3 out of the 6 moderators (see Table 1) do not appear on the top 10 list in Figure 3. One of the possible reasons for this is that some moderators may participate only in their own weekly chat and not be active during other weeks. From the community and knowledge building perspective, it would be beneficial to encourage moderators to participate in discussions moderated by others, especially prior to their own week. This would help the moderator to build authority in this community (encouraging more retweets), get to know what topics are important to this group, and be able to reference and connect to the topics discussed during the prior week(s).

### Prestige and In-Degree Centrality

As noted above, the total number of posted messages indicates only the engagement level on the part of an individual rather than the uptake of their contributions by the community. To find out whether personal messages influence others and make them reply or retweet, we examined *in-degree centrality* (the number of people who are mentioned or replied to). Table 2 shows the top 10 users based on in-degree centrality. Again, not surprisingly, the group founder is frequently mentioned by others, and her messages were retweeted by 36 people during the collected period of time.

In examining those on this list other than the founder, we noticed that they have something in common. Most of them have a very active online presence in social media in general, not just in this community. They are also very passionate and active commentators on health matters on Twitter. For example, the second most connected account is @cmaer. This username belongs to Pat Rich, who is an online editor for the Canadian Medical Association and has over 1000 followers. Shirley Williams (@williampearl) is a new media enthusiast and advocate at Strategic Leadership Forum and has over 3000 followers. Others on this list also have a considerable number of followers. In fact, there is a weak, monotonic (nonlinear) positive correlation between the number of followers and the in-degree centrality (Spearman rho=0.23, *P*<.01). In other words, people with more followers on Twitter in general are likely to be more central (based on the in-degree centrality) in this community.

One possible explanation of this could be that by participating in weekly discussions on #hcsmca, these individuals expose their followers to this community through their tweets on this topic (with the #hcsmca hashtag). As a result, their followers may also join #hcsmca chats and retweet or reply to them on this topic, thus increasing their in-degree centrality in this community. Future research is required to confirm or reject this preliminary supposition. If it holds, then one recommendation for growing an online community such as #hcsmca could be to find people who (1) are already actively engaged in online conversations in this area and (2) have a strong base of followers, and invite them to join the discussion. This is a reasonable recommendation in general as it brings in people who can act as bridges between separate networks and communities. People with high in-degree on this list are also good candidates for moderating future discussions as their messages are clearly resonating with this group.

Another observation that we can make about this group is that 6 of 10 people with high in-degree also posted the most number of tweets to this community (see Figure 3). This may just be indicative of their general interest in this topic but may also suggest that because they post more messages, their messages are more likely to be noticed by others on Twitter and thus more likely to be retweeted. Future research is needed to explore this further.

Overall, people with high values of in-degree centrality can be considered as trusted information sources whose opinions and comments are recognized as having value for the community, as evidenced by the frequency with which their messages are retweeted or they are frequently mentioned by others. These people are important for this community as they generate a lot of trusted, “sharable” information that generates discussion, but equally important, by being retweeted, also sustains conversational interaction and the life of the #hcsmca Twitter community.

**Table 2:** Top 10 users by in-degree centrality.

Twitter handle	Centrality in degree	Centrality out degree	Twitter profile description (as posted by the user)
colleen_young	36	27	Community Manager of Virtual Hospice | Portail en soins palliatifs (@VirtualHospice), Founder of #hcsmca, plain language writer, health literacy advocate
cmaer	33	10	Online editor for the Canadian Medical Association. Views are my own
williampearl	26	4	Facilitating & Finding Pearls in Strategy, SocialMedia & Healthcare, #ROTPt
naheedd	18	12	Medical resident physician. #GlobalHealth+#SDOH advocate. #MedEd+#hcsmca enthusiast. RA @CRICH_StMikes. Writer @HealthyDebate. Humanist. Change agent. Optimist.
infoway	17	1	News & announcements from Canada Health Infoway. Tweets by a team from Infoway. Check out our blog.
rdjfraser	15	1	Nurse & Author. Digital Tool Strategist and Educator. Learn more, help others. Tweets are my own.
anneccpa	14	10	Canadian Certified #PhysicianAssistant, practicing in #Orthopaedics - Sports Medicine & Trauma. Blogger & advocate for the Physician Assistant Profession.
alainabcyr	14	12	Learn, share, create. Grow. Aspiring expert in patient education and health communications on the Web and in print. Opinions are my own.
kathykastner	14	9	love listening learning sharing. appreciate humour happiness and eating chocolate covered almonds. End of life goal: a joyous exit. BestEndings.com
symplur	14	1	Connecting the dots in healthcare social media. Curator of Healthcare Hashtag Project; Social Media Consultancy

### Influence and Out-Degree Centrality

Another group of people who are important within any online community are people who monitor and retweet messages from others. To identify these individuals, we used the *out-degree centrality* (a measure of how often a person in the network mentioned or replied to other people in the network). Table 3 shows the top 11 users based on the out-degree centrality (11 users are shown rather than a more conventional “top 10” because of a tie in out-degree centrality for users who ranked 10th and 11th).

There is a strong overlap in who is prominent in both the in-degree and out-degree lists. Accounts such as @colleen_young, @naheedd, @alainabcyr, @cmaer, @anneccpa, and @kathykastner appear in both lists and thus are prominent because of both their in-degree and out-degree network connectivity. This shows their relative importance in this community as their messages resonated within the community (as indicated by their high in-degree centrality) and as they actively engaged others (as indicated by their high out-degree centrality). The remaining individuals on this list also have relative high values of the in-degree centrality (10 or more).

One anomaly is the community’s account @hcsmca. A review of its recent tweets reveals the account primarily posts announcements about upcoming Twitter chats for this community, mentioning Twitter handles of moderators and other special guests, but with little follow-on interaction with others. This suggests a potential method for identifying such accounts in order to exclude them from analyses of social networks: stark differences between in-degree and out-degree centrality may indicate a non-human, or non-community participant within a conversation.

**Table 3:** Top 11 users ordered by out-degree centrality.

Twitter handle	Centrality in degree	Centrality out degree	Twitter profile description
colleen_young	36	27	Community Manager of Virtual Hospice | Portail en soins palliatifs (@VirtualHospice), Founder of #hcsmca, plain language writer, health literacy advocate
natricer	11	22	retired PSW, family caregiver, passionate about elderly/vulnerable #hcsmca #dwdchat #eolchat #eldercarechat #caregivingchat #HCLDR #theWalkingGallery
naheedd	18	12	Medical resident physician. #GlobalHealth+#SDOH advocate. #MedEd+#hcsmca enthusiast. RA @CRICH_StMikes. Writer @HealthyDebate. Humanist. Change agent. Optimist.
alainabcyr	14	12	Learn, share, create. Grow. Aspiring expert in patient education and health communications on the Web and in print. Opinions are my own
samdunsiger	12	12	Freelance writer. Communicator. Volunteer #PR director for @SOSheadoffice, @stuttersocial. Caffeine and sushi addict.
hcsmca	0	11	Health Care Social Media Canada #hcsmca hosts a tweet chat every Wednesday at 1 pm EST (2 pm AST, noon CST, 11 am MST, 10 am PST).
cmaer	33	10	Online editor for the Canadian Medical Association. Views are my own
anneccpa	14	10	Canadian Certified #PhysicianAssistant, practicing in #Orthopaedics - Sports Medicine & Trauma. Blogger & advocate for the Physician Assistant Profession.
craigtyyz	13	10	Communications professional: digital, online and social media specialist at Women’s College Hospital. Views expressed are my own and not those of my employer.
kathykastner	14	9	love listening learning sharing. appreciate humour happiness and eating chocolate covered almonds. End of life goal: a joyous exit. BestEndings.com
quintepediatric	10	9	We provide medical care to infants, children and adolescents. Healthy kids energize our community! Our account is managed by Sara.

### Actor Roles

In the second part of the analysis, we were interested in learning more about the professional composition of this community and whether *professional roles* affect an individual’s position in the network. To address this, we first manually classified each Twitter user in the dataset into one of 11 roles (see Table 4). The classification was based on information in the user’s public Twitter profile. If information provided on Twitter was not sufficient, we followed links to the user’s personal website or LinkedIn page (if provided in their Twitter profile). For the purposes of analysis, users with multiple professional roles were listed as whichever they listed first in their own self-description.

**Figure 4:**
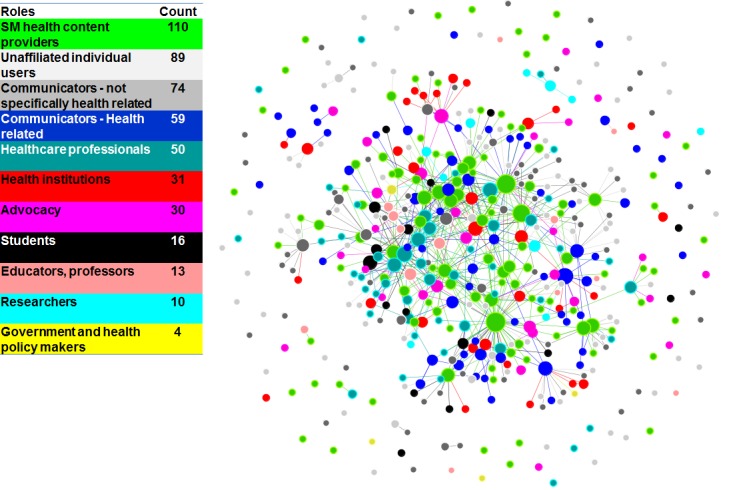
Twitter communication network on #hcsmca colored by professional roles, nodes sized by in-degree centrality.

Figure 4 shows the distribution of professional job classifications. The majority of participants in #hcsmca fit the category of “Social media health content providers”, describing themselves as dedicated to health topics and/or social media groups with a stated purpose of spreading health information. The second largest group was “Communicators”, but not those exclusively focused on health. These were mostly social media marketers whose relation to the network seemed topical or client-based. The third largest group was health-related “Communicators”. Although the first three groups were providers of social media health content or communicators, generally speaking, the #hcsmca network is relatively diverse as it also includes a number of health professionals, health institutions, advocacy groups along with health students, educators, and others. The smallest group, with only 4 representatives, was “Government and health policy makers”.

Absolute counts of the number of members in a particular professional group do not necessarily reflect the importance of any particular professional group in the network. Thus, to see whether any particular group was especially important in this network, an analysis of variance was conducted comparing in-degree centrality by group. We found a statistically significant relationship between professional roles and in-degree centrality (explaining about 7% of the variance, *P*=.003, using 5000 permutations), indicating that some professional groups are more influential in this community.

Next, we attempted to determine which professional groups were more or less likely to influence discourse in this group. Based on the average in-degree centralities for each of the 11 professional groups (see Table 5), social media health content providers were the most influential group with an average in-degree centrality of 2.89. (Notably, this group is also a clear leader based on the average out-degree centrality.)

The importance of this group in this network can also be visually observed in the graph representation of this community in Figure 4. In this graph, each node represents a Twitter user in this community, and the line connecting any 2 nodes means that there was at least one mention or reply between the 2 users in the network. This network graph shows that social media health content providers (displayed in the light green color) occupy key central positions in this network and often play a bridging role connecting members from other clusters of this network.

Interestingly, although there are fewer health professionals, educators, and health institutions in this community, their average in-degree centrality came in second, third, and fourth (see Table 5), indicating their relative importance in the network. By contrast, communicators, regardless of their strong presence in this network, were not as central as a group as the three groups just mentioned (despite a few nodes that appear to be in a star-configuration in this network). This may suggest that perhaps communicators are participating in this community because it is part of their job description, but they may not have a lot to contribute, and/or they are there to learn more about this subject matter and are tasked with reporting what they find back to their organizations. Future research is needed to explore this.

Another important observation is that although there seems to be a relationship between professional role and in-degree centrality, there is no apparent preferential attachment among people in the same professional group. In other words, the formation of connections among community members is not necessarily constrained by their professional status. This finding was supported by an analysis of variance density test using both the Structural Blockmodel technique (it examines “whether the different classes have significantly different interaction patterns”), and also with the Variable Homophily model (which “assumes that each group or class of actors has a different homophilic tendency” [42]; where homophily is the tendency for connection based on social similarity). Based on this test (run with the 5000 permutations), the professional roles explain only 0.2% of the total variance (*P*=.005) when run with the Structural Blockmodel and only 0.1% (*P*<.001) with the Variable Homophily model. This result indicates connections are more prevalent across members with different professional backgrounds and occupations in this community, which in turn may suggest that this is a welcoming environment that stimulates knowledge exchange and learning across professional boundaries.

**Table 4:** Professional roles.

Category	Sample profile of a Twitter user classified under this category
Advocacy	@PatientsAssocCa - The Patients’ Association of Canada promotes the role of the patient in all areas of health care. Follow: Donate:
Communicators—health related	@Infoway - News & announcements from Canada Health Infoway. Tweets by a team from Infoway.
Communicators—not specifically health related	@bobbigreenberg - Dynamic communications & public affairs consultant. Mentor & Coach. Passionate about learning new languages, travel, teaching yoga. Stop, pause and breathe.
Educators, professors	@jendlake - Assistant Professor & Pharmacist. Collaboration/ communication will improve patient-care. Tweets are mine and include primary care, medications and good food
Government and health policy makers	@healthcouncilca - The Health Council of Canada reports on the progress of health care renewal and on innovative practices in Canada.
Health institutions	@QuintePediatric - We provide medical care to infants, children and adolescents. Healthy kids energize our community! Our account is managed by Sara.
Health care professionals	@DrJenGunter - OB/GYN, writer, sex health expert, defender of evidence-based medicine. I wield the lasso of truth. Tweets are not medical advice. I speak for no one but me.
Researchers	@CBoC_HIPE - Independent, leading-edge policy research from the Health Innovation, Policy and Evaluation team at the Conference Board of Canada.
Social media health content providers	@HeartSisters - On women & heart disease from the unique perspective of Carolyn Thomas, a Mayo Clinic-trained heart attack survivor/women’s health advocate. Also
Students	
Unaffiliated individuals	@JEANIESBEACH - music, dance; fashion, women’s rights

**Table 5:** Average centrality per professional group.

Role	Average in-degree centrality	SD	Average out-degree centrality	SD
Social media health content providers	2.89	6.17	2.21	3.96
Health care professionals	2.48	4.45	1.86	2.28
Educators, professors	2.00	2.97	1.31	1.65
Health institutions	1.65	2.51	1.23	1.71
Advocacy	1.47	2.50	1.10	1.18
Communicators—Health related	1.39	3.03	1.32	1.31
Students	1.38	2.68	1.88	2.03
Researchers	0.90	1.45	0.90	0.99
Government and health policy makers	0.75	1.50	0.50	0.58
Communicators—not specifically health related	0.68	2.18	1.34	1.67
Unaffiliated individual users	0.08	0.34	1.06	1.11

## Discussion

### Principal Findings

This and previous studies in this area have highlighted how online communication extends the possibilities of community, that is, bringing participants together to form communities of interest for those geographically remote from one another [45]; augmenting geo-community through online information and forums for interaction related to local events and conditions [46]; and extending interaction times and methods through online/offline combinations, as in new forms of online and blended learning, and in the way our communications (text, email, Internet) cross devices (phones, tablets, computers) and contexts (home, work, office). New analytical techniques also push the definition by discovering community on the basis of online interaction, suggesting new definitions and considerations around what constitutes community and what criteria we will accept for identifying it [17].

The current study highlighted some initial observations of the structure of the community formed around the #hcsmca hashtag. As has been asked in the past, how can a group of individuals who meet online, through the lean medium of Twitter, and the constraints of a 140-character posting, sustain and be considered a community? Results from our limited sample set suggest this has happened through a strong core of active participants including the group founder, who lead in posting and prominence in the network. Attention to others is an important aspect of community, and the measures of influence and prominence presented here show that attention to others in the group exists, with key players recognized through mentions and retweeting. The configuration of the community and communication by role suggests one major component connecting all participants, that is, the conversation is not fragmented into isolated cliques. Weekly discussions provide a boost to interaction that stimulates activity and provides a dependable rhythm to interaction patterns and a site to return to each week.

Many studies of community and online community have taken place before ours. The following are some of the observations derived from the results of this and related studies, with commentary on the impact in relation to the #hcsmca community studied here.

### Leadership

Some notable attributes of community reported from many studies and associated with both online and offline collectives are local language, shorthands and in-group signifiers [47,48]; group-defined genres, rules of conduct, and policing of conduct [47,49-53]; and interpersonal self-disclosure, emotional support, and shared history (eg, [2,54-58]). In their joint definition of behaviors and practices, there is also attachment to aspirations for a shared future, for example, in group adoption of shared goals and missions, or in the expectation that practices as they exist will be honored and valued in the future. Shared expectations about future commitments enhance trust in the community and its members [59].

In #hcsmca, the very use of Twitter is the community genre, shorthand, and local language. The weekly discussion gives promise of a shared future, as does the general attention to issues relating to health care in Canada and working in this area. While an analysis of tweet content is necessary to discover more about the relations connecting individuals, retweets have provided evidence of attention to others’ comments and thus an orientation to community members.

Developing community further depends on continuing attention to the kinds of outcomes that have been found to characterize community, both by design and by emergence from community interaction. Earlier work on online communities and virtual teams has revealed the way rules and norms emerge and evolve with community interaction, with the direction of emergence depending on both technological affordances and the salience to participants of social, informational, and technical features [50,60,61]. Others have noted the need for initial contribution by altruistic or proactive communicators who build the critical mass of participants and participatory interactions [62,63] and create the “safe space” for interaction [49]. However, communities need to move on from these key communicators or risk the demise of the community when such actors leave [44].

Altruistic, proactive use by the #hcsmca founder and by core users remains an important feature for building this community. In looking to the future, the community may face opportunities and challenges in incorporating more and new technology into its repertoire as it expands and as new needs arise that extend the reach and scope of the community. In each round of such expansion, core participants may again have to lead and stimulate contribution and participation as they help develop the character of their community.

### Participation

Along with leading a community, there is also the experience of those who lurk, listen, join, participate, and depart from communities. Joining an online community is much like joining any community in the need to learn the norms of behavior, the language used, and who is who among members. Online, this is accomplished through observation and (usually) text-based communications. Joining entails phases. Studies of online learning communities revealed stages of *joining*, *maintaining presence*, and of *disengaging* from the online community [56,64]. Joining can entail learning the norms of the environment, for example, learning how to express oneself in the 140 characters of a tweet. Joining often entails a stage of observation, for example, reading but not posting to online conversations.

It is still a question of how, and why, community can be formed and sustained via text-based communications. Early opposition to the notion of “virtual community” pointed to the lack of nuance of face-to-face interaction and the “leanness” of text as a basis for interaction. Critics noted difficulties in conveying tone, emotion, intimacy, and complex information, and the lack of personal identity and accountability with anonymous participants or the use of pseudonyms (online “handles”). Yet, online communicators found ways around these shortcomings, quickly and easily adopting means of conveying information, enjoying their anonymity, and expressing emotion through texts (eg, with emoticons). Early explorers of these new regions were able to observe the reformation of social and communal ties through online means as these were “uncoupled” from face-to-face interaction (eg, [14-16,45,47,56,65]).

Yet, another effect observed for these lean media has been the reduced inhibitions associated with communicating, for example, the ability to talk through text without face-to-face contact or the need for immediate response. This can be an important feature that encourages new career professionals to communicate (as in #hcsmca), or patients to discuss emotional experiences [2,8,9].

Although our analysis did not focus on newcomers, the results and overall structure of the network suggests that a significant number of isolates, not connected in the Name Networks that signify attention to others (see Figure 2). Such lurkers, while often considered negatively, can also be at the positive stage of what Lave and Wenger termed *legitimate peripheral participation* when new, potential members learn and immerse in the norms and knowledge of the community [43]. However, an overabundance of lurkers can put posters on the spot, inhibit the communal aspect of the site, and fail to create the interactivity necessary for long-term viability [66]. Moreover, while all participants may benefit, individuals may benefit less when lurking rather than participating, as found in a study of a breast cancer support group [5]. Thus, it is important to have new members take up the conversation and participate. Where #hcsmca leaders can become aware of what motivates these isolates, it may help to understand how to support their greater presence in the community.

### Online/Offline Synergies

While social media may be considered in their online context only, it has long been recognized that media are not used in isolation from offline interaction and that they are instead embedded in everyday life [67]. This is even more true today as mobile devices, wireless networking, and mobile phone connections afford communication anywhere, anytime [68,69]. We weave and juggle social, learning, and work interactions across media, and across home, school, and work boundaries [46,70-72]. Similarly, while social media may be considered one at a time, relationships, communities, and information behaviors are more often maintained through various media. Several studies have shown that those who maintain closer ties use more media to communicate [73], that is, those who have a greater need or desire to communicate use more of the available media to do so. More forms of interaction, for example, through multiple connections to others, can also increase the value of engagement. This can include using more features of a site: Web access logs of the use of the site PatientsLikeMe, showed those who used more features perceived greater benefit from using the site [7].

Media are not used in isolation but as part of a repertoire that affords connection to resources and to others. This repertoire also includes face-to-face interaction and can support blended learning [70] and blended health [6]. Online interaction provides the opportunity for continuing interaction, learning and care across specializations, disciplines, institutional venues, and structured meeting times. The media then become a tool to facilitate patient-centered, collaborative care [6].

Moreover, it is not just delivery of information that is involved in these collaborations. From considerations of community, attention has expanded along with new forms of social media to consider different forms of interaction, from the friend relationships of Facebook and other social networking sites [11,74,75] to the benefits and interaction patterns associated with participatory culture, peer production [9,76-79], and crowdsourcing for open collaborations and commercial applications [7,8,10,80-85].

Our analysis of HCSMCA did not focus on the multiple platforms that participants use for health care information and conversation, or the way #hcsmca fits with other parts of participants’ lives. As such, results about community based solely on Twitter interaction have the potential to underestimate the foundations for community that come from joint and shared interaction across networks and platforms. Again, this is something worthy of further analysis and of interest to community builders as they consider how Twitter works with other venues to help support their community.

### Conclusions and Future Directions

We asked at the outset what accounts for the relative longevity of this particular online community and have found that it is based on interaction patterns of participation and prominence of the group’s founder and a small core of key participants who are heavily engaged in social media and health care networks. Longevity is supported by the structure of weekly discussions, which creates a communal structure for interaction. We also asked what constitutes the composition of this community and found that it consists of individuals who may be classified as belonging to a number of different roles, but that communication flows across roles and thus the network reveals a community of one major component. The network also shows a large number of individuals who are present in the community but not actively connected to others and who may be benefitting from observation of the conversations and are potential future active participants.

Our purpose has been to show how a social network analysis can reveal such patterns and how past work on online community can help in interpretation of such results for the creation and maintenance of online communities for health. In brief, the implications and recommendations are:

- Leaders and core participants can seed a network by altruistic or proactive use that, initially, provides more benefit to others than they receive in return. However, for long-term sustainability that persists beyond leadership change, the network needs to grow in a way that distributes leadership and participation beyond single leaders.
- Prominence in the network appears to be related to familiarity with individuals, for example, more active participants receive more attention in terms of mentions and retweets. Thus, a recommendation is that moderators of discussions build authority in the network prior to their moderation duties to be able to connect better with ongoing discussions.
- More prominent actors are engaged in multiple networks relating to health matters. As these actors also bridge networks, they are able to carry the message of the network to others. Thus, a recommendation is to engage these types of actors as a way of increasing the reach and prominence of the network itself.
- Peripheral participants represent untapped resources for the network. Finding out what motivates such participants can help identify those who will make contributions in the future and thus how to bring their participation into the community.
- Network analysis and visualizations provide a set of techniques and a vocabulary about network interactions that can help both group leaders and participants to see the size, shape, and configuration of the network in order to gain a better understanding of its operation and the place of individuals in that operation. Attention to roles can reveal both emergent roles (eg, core participants) as well as show the influence of existing roles (eg, different medical or sector roles).

This analysis of one social media site highlights the way social network analysis can be used to gain an understanding of social media use for communication and conversation and how network formations support such communities. However, this example has barely covered the beginnings of potential applications. Some key questions that remain and can form the basis of future work are: How do we implement and measure the impact of social media on health for individual patients and for the general population? What single and/or combination of media provide interaction around health that is effective over the short and long term? What combinations of participation and contribution create interest and sustain communities that discuss and continue to apply better practices for health and well-being? The task is complex as it requires understanding the rapidly changing and expanding media options in relation to changing institutional and societal practices, yet the opportunity is there.

We believe the principles of social networks and the techniques of social network analysis provide a solid foundation for understanding relationships and their formation online and for taking that into social media practice for health. Attention to network relations emphasizes what we do together, rather than what medium or face-to-face venues we use. This approach has proved useful for understanding the societal turn to online communication, relational maintenance, community genesis, and sustainability. There are already studies and models that have addressed health from a social network perspective (eg, [12,13,19]), and there is much that has addressed online interactions from a social network perspective (eg, [17,86]). As we turn to considering their interaction and the specific application of social media for health (eg, [5,9,11,25]), we look forward to combining these to explore further the interplay of social media, social networks, health, and well-being [8,10,87].
